# 2D Gait Skeleton Data Normalization for Quantitative Assessment of Movement Disorders from Freehand Single Camera Video Recordings

**DOI:** 10.3390/s22114245

**Published:** 2022-06-02

**Authors:** Wei Tang, Peter M. A. van Ooijen, Deborah A. Sival, Natasha M. Maurits

**Affiliations:** 1 Department of Neurology, University Medical Center Groningen, University of Groningen, P.O. Box 30001, 9700 RB Groningen, The Netherlands; n.m.maurits@umcg.nl; 2 Data Science Center in Health, University Medical Center Groningen, University of Groningen, P.O. Box 30001, 9700 RB Groningen, The Netherlands; p.m.a.van.ooijen@umcg.nl; 3 Department of Pediatric Neurology, University Medical Center Groningen, University of Groningen, P.O. Box 30001, 9700 RB Groningen, The Netherlands; d.a.sival@umcg.nl

**Keywords:** early onset ataxia (EOA), developmental coordination disorder (DCD), AlphaPose, gait, pose estimation

## Abstract

Overlapping phenotypic features between Early Onset Ataxia (EOA) and Developmental Coordination Disorder (DCD) can complicate the clinical distinction of these disorders. Clinical rating scales are a common way to quantify movement disorders but in children these scales also rely on the observer’s assessment and interpretation. Despite the introduction of inertial measurement units for objective and more precise evaluation, special hardware is still required, restricting their widespread application. Gait video recordings of movement disorder patients are frequently captured in routine clinical settings, but there is presently no suitable quantitative analysis method for these recordings. Owing to advancements in computer vision technology, deep learning pose estimation techniques may soon be ready for convenient and low-cost clinical usage. This study presents a framework based on 2D video recording in the coronal plane and pose estimation for the quantitative assessment of gait in movement disorders. To allow the calculation of distance-based features, seven different methods to normalize 2D skeleton keypoint data derived from pose estimation using deep neural networks applied to freehand video recording of gait were evaluated. In our experiments, 15 children (five EOA, five DCD and five healthy controls) were asked to walk naturally while being videotaped by a single camera in 1280 × 720 resolution at 25 frames per second. The high likelihood of the prediction of keypoint locations (mean = 0.889, standard deviation = 0.02) demonstrates the potential for distance-based features derived from routine video recordings to assist in the clinical evaluation of movement in EOA and DCD. By comparison of mean absolute angle error and mean variance of distance, the normalization methods using the Euclidean (2D) distance of left shoulder and right hip, or the average distance from left shoulder to right hip and from right shoulder to left hip were found to better perform for deriving distance-based features and further quantitative assessment of movement disorders.

## 1. Introduction

Children with Early-Onset Ataxia (EOA) and Developmental Coordination Disorder (DCD) both show features of motor incoordination, hampering clinical distinction [1]. Gait analysis plays a pivotal role in motor coordination assessment, contributing to early diagnostics and rehabilitation [2]. In the clinical context, ataxic gait can be assessed by validated, semi-quantitative rating scales such as the Scale for the Assessment and Rating of Ataxia (SARA) [3]. Despite the high reliability of the clinical scales, evidence shows they might be less effective in measuring diverse motor control strategies and/or mixed disorders [4]. Moreover, their application may depend on the evaluation and interpretation of experts, especially in children with mixed movement disorder features [5,6]. Altogether, usage of clinical rating scales such as the SARA can sometimes be regarded as subjective, sensitive to confounding factors and time-consuming [4].

Inertial wearable sensors can provide an objective and feasible alternative for gait assessment [7]. The use of inertial measurement units (IMUs) with accelerometers and gyroscopes linked to the body with elastic straps has been investigated as an aid in the differential diagnosis of early onset ataxia and developmental coordination disorder [8]. Automatic classification based on quantitative gait features from IMUs has been shown to outperform phenotypic diagnosis, implying that movement quantification and subsequent automatic classification might be a useful technique for consistent and repeatable diagnostic evaluation [9]. However, this approach requires special preparation before use, and one of the problems to overcome is how to measure distance-based features such as step width, which is crucial for diagnosing ataxia: ataxia patients walk with reduced step length and increased walking base width [10].

Human pose estimation is one of the fundamental tasks in computer vision. This task can often be subdivided into single-person and multi-person pose estimation, as well as 2D and 3D pose estimation, depending on whether the video or image dataset contains multi-person or 3D depth information. Owing to the fast development of deep learning, many convolutional neural network (CNN) based frameworks have been designed and published, such as Maskr-cnn [11] DeeperCut [12], DeepLabCut [13], CPN [14], OpenPose [15], which use convolutional pose machines to first locate human joints in an image and then a part affinity field to complete the human body assembly, and AlphaPose [16], which contains a symmetric spatial transformer network, parametric pose no-maximum- suppression, and a pose-guided proposal generator. Currently, the applications of pose estimation include motion capture for actors [17], assessment of athletes [18], and fall detection [19]. Compared with other frameworks, AlphaPose can achieve higher comprehensive accuracy on the MSCOCO dataset [20] with a map value of 72.3 [16], leading to its application in video behavior detection [21], action recognition [22], and gait analysis [23,24,25,26].

In the daily clinical setting, neurologists routinely record patients’ movements for further evaluation. The ubiquity of video cameras in outpatient clinics and recent advances in video-based pose estimation have motivated us to design a 2D skeleton-based method for quantitative analysis of movement disorders using video images taken in the coronal plane. In a clinical setting, sagittal plane video recordings are typically not available, due to space limitations. Through pose estimation, the joint coordinates of the patient in single camera video recordings can be obtained in a convenient and low-cost way. The use of 2D video-based motion capture for gait analysis dates back to the last century [27]. It has so far covered a wide range of topics, from disease diagnosis to rehabilitation; several of those contributions relied on AlphaPose [23,24,25,26,28]. Mehdizadeh et al. [28] measured gait parameters in elderly adults and discovered that temporal, but not spatial or variability gait measures, derived from AlphaPose correlated with those calculated with a motion capture system in the frontal plane. Sabo et al. [25] made predictions of parkinsonian gait in older adults with dementia and for estimating parkinsonian severity using natural gait videos of older adults [26]. Peng et al. studied differences in gait parameters between the healthy population and patients with lower extremity dyskinesia [24]. Lv et al. found significant differences in the entropy of heel and ankle joint motion signals between healthy people and arthritic patients, which could be used to identify patients with knee arthritis [23]. To allow the calculation of changes in distance-based features, we need to normalize these 2D coordinates first. However, to the best of our knowledge, no prior study has discussed how to perform normalization to derive distance-based features.

Here, we propose and compare seven different normalization methods that enable the calculation of distance-based features derived from pose estimation results. The normalization method needs to address two issues: (1) the skeleton size varies depending on the distance from the camera in every frame, (2) the skeleton position in each frame might change due to camera instabilities. These problems induce nonphysiological frame-to-frame variability, hindering calculation and comparison of distance-based features. We here initially apply the AlphaPose deep learning model and PoseFlow framework to obtain skeleton keypoint data from ataxia, DCD and the controls. We anticipate that the best normalization method will maintain the constancy of certain distances in the coronal plane, such as the distance between the two shoulders when walking, while maintaining the variability of other distances, such as the distance between the two wrists.

## 2. Materials and Methods

### 2.1. Pipeline

**Figure 1:**
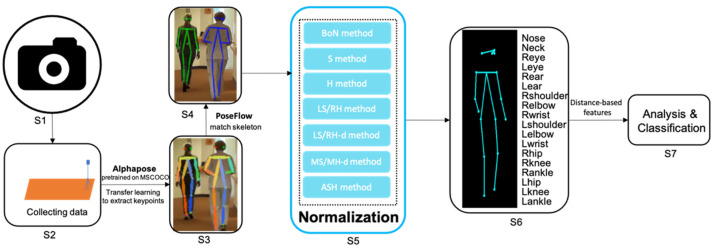
Overview of the proposed pipeline for the quantitative assessment of gait from freehand 2D camera recordings. From left to right: S1: Configure the camera and its settings. S2: Collect data with the camera. S3: Extract keypoints using Alphapose. S4: Match the skeleton using PoseFlow. S5: Normalize the skeleton data with one of methods given in Section 2.4. (BoN: box normaliztion; S: shoulder normaliztion; H: hip normalization; LR/RH: left-shoulder right-hip normalization; LS/RH-d: left-shoulder right-hip distance normalization; MS/MH-d: mid-shoulder mid-hip distance normalization; ASH: average shoulder hip normalization). S6: Obtain the normalized keypoint skeleton sequences. S7: Analyze the (distance-based) features derived from skeleton data for classification.

The entire pipeline for quantitative assessment of gait from freehand 2D camera recordings is summarized in Figure 1. A monocular camera is placed in front of the participant for recording. After the video data is obtained, a deep neural network model pretrained on the MSCOCO dataset [20] based on AlphaPose [16] is used to extract skeleton keypoints. PoseFlow [29] is applied to match the skeleton to the same participant in a recording. The keypoint data are then normalized using one of the proposed normalization methods, allowing distance-based features derived from the scaled 2D trajectories to be compared across different movement disorders. Finally, these extracted features can be used to perform quantitative analysis or classify participants. The details are explained below.

### 2.2. Data Preparation

The data for this study were collected at the University Medical Center Groningen (Groningen, The Netherlands), in accordance with local research and integrity codes. Fifteen children, including five with EOA (mean age 12.6 years, SD 1.85 years), five with DCD (mean age 9.6 years, SD 3.21 years), and five healthy controls (mean age 9.4 years, SD 1.81 years) participated in the experiment. All included children older than 12 years of age and all parents of the children included provided informed consent before study enrollment. Children younger than 12 years of age also provided informed assent. EOA patients had radiologic (MRI), metabolic, and laboratory and genetic testing at the department of (pediatric) neurology at the University Medical Center Groningen as part of their diagnostic evaluation. DCD patients were also evaluated at the pediatric neurology outpatient clinic. When applicable, these evaluations included MRI, electromyography, muscle ultrasound, and/or laboratory and sometimes also genetic testing to exclude other underlying neurologic disorders.

Children were asked to walk freely and repeatedly in a straight line while an experimenter stood at the end of the corridor holding a single 2D video camera (1280 × 720, 25 fps) facing the subjects for recording. They were asked to walk at their own pace in a corridor of approximately 15 m, make a 180 degree turn and return to the starting position, in accordance with the SARA regular gait task guidelines. EOA was clinically confirmed in accordance with its definition [30]. For further processing, each video recording was divided into segments belonging to the following categories: (1) walking towards the camera, (2) walking away from the camera, (3) standing still, (4) turning around. We only analyzed the video segments of walking towards and away from the camera. In addition, frames in which body parts were occluded or lost were excluded from further analysis, because the skeletons in these frames could not be detected correctly using the current 2D pose estimation algorithm. In total, 60 segments of video data (15 participants, two types of gait, two segments each) were used for skeleton extraction.

### 2.3. Pose Estimation

After cleaning the video data, we used AlphaPose, a deep neural network model, to estimate the 2D locations of skeleton keypoints. Here, we utilized the Yolo-v3 detector [31], pretrained on the MSCOCO dataset, to detect people in the video frame. Subsequently, the fast resnet50 [32] model was chosen to obtain the final locations of each keypoint. For each frame, the model returned 2D coordinates (in pixels) as well as a prediction confidence probability for each of the 17 keypoints of each person detected, including the nose, and bilateral eyes, ears, shoulders, elbows, wrists, hips, knees, and ankles. Finally, we used a tracker, PoseFlow, to match the skeleton to the same participant in each recording.

### 2.4. Normalization

We proposed and analyzed different normalization methods. Here, the original keypoint data of each video segment were presented as a sequence of *N* continuous skeletons (*S*_1_, *S*_2_, *S*_3_*… S_N_*), where skeleton *Si* of the *i*-th frame (*i* = 1… *N*) is composed of 17 pairs of coordinates (in pixels) representing all 17 keypoints.

It should be noted that movement inside the coronal plane (such as up-and-down or side-to-side movement of a limb) has no effect on distance computation, but movement outside the plane (such as walking towards or away from the camera, or body rotation) does. For this reason, we consider both the Euclidean distance between two joints and the distance between two points in the horizontal and vertical directions, respectively, for the different normalization methods. We consider two-step solutions, that include position shifting and size scaling.

Step 1: *Position shifting*. This method achieves the result that the mid- shoulder point becomes the origin (0,0) in each frame, by shifting all coordinates according to:

(1) $$X_{S_{i}}{}^{\prime }=X_{S_{i}}-\frac{x_{S_{i}}^{\mathrm{Ls}}{+x}_{S_{i}}^{\mathrm{Rs}}}{2\;},\;Y_{S_{i}}{}^{\prime }=Y_{S_{i}}-\frac{y_{S_{i}\;}^{\mathrm{Ls}}{+y}_{S_{i}}^{\mathrm{Rs}}}{2\;}$$

Here ($X_{S_{i}}{}^{\prime },Y_{S_{i}}{}^{\prime }$) are the position-shifted coordinates in the skeleton, $S_{i}$, ($X_{S_{i}},Y_{S_{i}}$) are the original coordinates and ($x_{S_{i}}^{\mathrm{Ls}},y_{S_{i}\;}^{\mathrm{Ls}})$ and ($x_{S_{i}}^{\mathrm{Rs}},{\;y}_{S_{i}}^{\mathrm{Rs}}$) are the coordinates of the left shoulder and right shoulder in the skeleton $S_{i}$, respectively.

Step 2: *Size scaling*. Seven methods were considered that each used different distances to scale every video frame, according to:

(2) $$X_{S_{i}}{}^{\prime \prime }{=w}_{0}\;*\;\frac{X_{S_{i}}{}^{\prime }}{w_{S_{i}}\;},\;Y_{S_{i}}{}^{\prime \prime }{=h}_{0}*\;\frac{Y_{S_{i}}{}^{\prime }}{h_{S_{i}}\;}$$

Here ($X_{S_{i}}{}^{\prime \prime }$,$\;Y_{S_{i}}{}^{\prime \prime }$) and ($X_{S_{i}}{}^{\prime }$,$\;Y_{S_{i}}{}^{\prime }$) are the scaled and position-shifted coordinates, respectively, and $w_{S_{i}}$ and $h_{S_{i}}$ are width and height defined by the specific normalization method. Four of the assessed normalization methods use the width and height of the bounding boxes of: (1) all keypoints (Box scale or BoN method), (2) the two-shoulder keypoints (S method), (3) the left-shoulder and right-hip (LS/RH method) and (4) the two-hip keypoints (H method). The other methods use the Euclidean (2D) distance between (5) the left shoulder and right hip for both w and h (LS/RH-d method) and (6) the mid shoulder and mid hip for both w and h (MS/MH-d method). The last method uses the average distance from the left shoulder to right hip and from the right shoulder to left hip for both w and h (ASH method).

Finally, ($w_{0}$,${\;h}_{0}$) can be obtained by averaging the width $w_{S_{i}}$ and height $h_{S_{i}}$ of each video segment, according to:

(3) $$w_{0}=\frac{1}{N}\sum_{i=1}^{N}w_{S_{i}},\;h_{0}=\frac{1}{N}\sum_{i=1}^{N}h_{S_{i}}$$

To visualize and reconstruct the video after normalization, we finally shift the whole skeleton to the middle of the image:

(4) $$X_{S_{i}}{}^{\prime \prime \prime }=X_{S_{i}}{}^{\prime \prime }+x_{0},\;Y_{S_{i}}{}^{\prime \prime \prime }=\;Y_{S_{i}}{}^{\prime \prime }+y_{0}$$

Here, $x_{0}$, $y_{0}$ are constant values; we used $x_{0}\;$ = 640 and $y_{0}\;$ = 200 as we have a resolution of 1280 × 720.

(1), (2) and (4) together can be written as follows:

(5) $$X_{S_{i}}{}^{\prime \prime \prime }=\frac{w_{0}}{w_{S_{i}}\;}\;(X_{S_{i}}\;-\;\frac{x_{S_{i}}^{\mathrm{Ls}}{+x}_{S_{i}}^{\mathrm{Rs}}}{2\;})+x_{0}$$

(6) $$Y_{S_{i}}{}^{\prime \prime \prime }=\frac{h_{0}}{h_{S_{i}}\;}\;(\;Y_{S_{i}}\;-\frac{y_{S_{i}\;\;}^{\mathrm{Ls}}{+y}_{S_{i}}^{\mathrm{Rs}}}{2\;})+y_{0}$$

**Figure 2:**
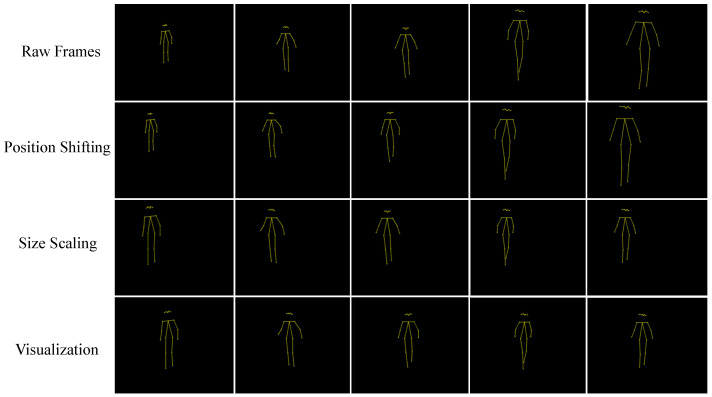
Example of several frames of an EOA patient walking towards the camera illustrating the effect of the different normalization steps when using the ASH method.

To illustrate the effect of the different normalization steps on skeleton size and position, we show several frames of an EOA patient walking towards the camera at each step of the normalization process in Figure 2.

### 2.5. Evaluation

Performance was assessed by calculating the mean value of the absolute error of the angle (mean absolute angle error) and the mean variance of the distance between certain keypoints. It is important to note that we cannot obtain any true physical distance measurements because we lost depth dimension information from the start by using a 2D camera. Therefore, our evaluation metrics are inspired by our clinical task, with the expectation that in the ideal case (1) there should be no changes in angles between vectors in the coronal plane before and after normalization, (2) normalization should maintain the variability of the distance between the two wrists and the two ankles, and (3) the constancy of the distances between the two shoulders and between the two hips is maintained (assuming that out-of-coronal-plane rotation is limited during gait towards and away from the camera). We selected four relative angles (angles between vectors): the angles between the left- and right-wrist–elbow vector and the elbow–shoulder vector, and the angles between the hip–knee vector and the knee–ankle vector. We also selected eight absolute angles (angles between vectors and the horizontal line) related to the left- and right-wrist–elbow, elbow–shoulder, hip–knee, and knee–ankle vectors. For comparison of the mean variance of distance, we selected the distance between the two shoulder, wrist, hip, and ankle keypoints, expecting that after normalization the variance of the distance between the two shoulder and between the two hip keypoints should be close to 0 (due to physical limitations), but the variance of the distance between the two wrist and two ankle keypoints should be as large as possible (expecting most variability in movement from these keypoints).

## 3. Results

### 3.1. Likelihood from Pose Estimation

Table 1 provides the mean and standard deviation of the likelihood (confidence probability) of the 17 keypoints for all participants and segments before and after cleaning. The average likelihood of skeleton keypoint locations obtained from pose estimation using raw video data was 0.817 (SD = 0.09). A paired-samples *t*-test was conducted to compare the likelihood of prediction of keypoint locations before data cleaning and after data cleaning. After noisy frames and the standing and turning around segments were removed, there was a significant increase of the probability (M = 0.889, SD = 0.02, t(28) = 3.36, *p* < 0.05).

### 3.2. Mean Absolute Angle Error

**Figure 3:**
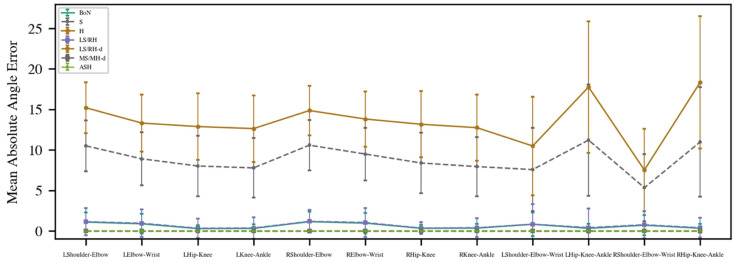
Mean absolute angle error results of proposed methods. (BoN: box normalization; S: shoulder normalization; H: hip normalization; LR/RH: left-shoulder right-hip normalization; LS/RH-d: left-shoulder right-hip distance normalization; MS/MH-d: mid-shoulder mid-hip distance normalization; ASH: average shoulder hip normalization).

The results for the mean absolute angle error are provided in Figure 3. The H method, especially for the eight absolute angles, has the largest error (M = 14.60), followed by the S method (M = 10.39). These two normalization methods had much higher errors compared with the other methods, hence they were omitted from further consideration. The other normalization methods had similar good performances.

### 3.3. Mean Variance of Distance

**Figure 4:**
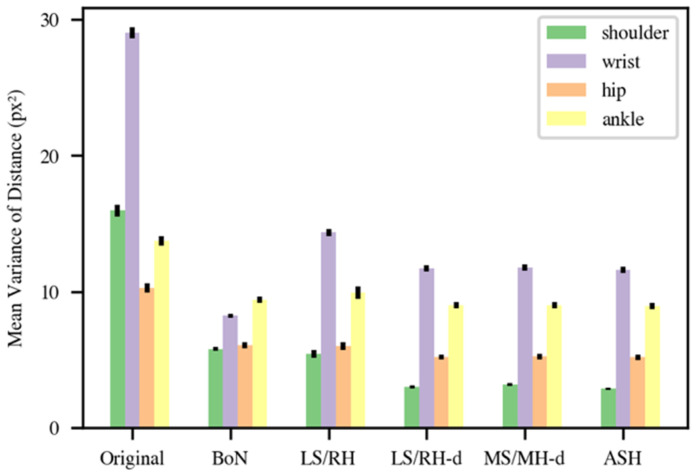
Mean variance of distance between shoulder (green), wrist (purple), hip (orange) and ankle (yellow) keypoints. (BoN: box normalization; LR/RH: left-shoulder right-hip normalization; LS/RH-d: left-shoulder right-hip distance normalization; MS/MH-d: mid-shoulder mid-hip distance normalization; ASH: average shoulder hip normalization).

Figure 4 presents the mean variance of the distance between shoulder, wrist, hip, and ankle keypoints for the original data and the BoN, LS/RH, LS/RH-d, MS/MH-d, and ASH methods. As expected, the mean distance variances were high (shoulder: 16.33, wrist: 29.35, hip: 10.29, ankle: 13.74) in the original data. The results of the BoN (shoulder: 5.67, wrist: 8.18, hip: 5.96, ankle: 9.24) and LS/RH (shoulder: 5.26, wrist: 14.08, hip: 5.83, ankle: 9.69) methods were similar, with the exception that LS/RH normalization produces somewhat more fluctuation in the wrist component. The final three normalization methods (LS/RH-d, MS/MH-d, and ASH) provided fairly similar results with minor differences. The results of MS/MH-d (shoulder: 3.08, wrist: 11.52, hip: 5.09, ankle: 8.81) were a little higher than those of LS/RH-d (shoulder: 2.90, wrist: 11.49, hip: 5.06, ankle: 8.79), but these differences were not significant (shoulder: U = 54, *p* = 0.16; wrist: U = 62, *p* = 0.29; hip: U = 57, *p* = 0.20; ankle: U = 68, *p* = 0.41; Mann–Whitney U-tests). Although the shoulder and hip variances of the ASH method obtained the lowest variances (shoulder: 2.78, wrist: 11.32, hip: 5.05, ankle: 8.77) among all normalization methods, there was no significant difference between the MS/MH-d and ASH methods either (shoulder: U = 69, *p* = 0.44; wrist: U = 68, *p* = 0.42; hip: U = 65, *p* = 0.35; ankle: U = 67, *p* = 0.40; Mann–Whitney U-tests). However, this method balanced using the distance between the left shoulder and right hip and the right shoulder and left hip to achieve a more stable performance.

**Figure 5:**
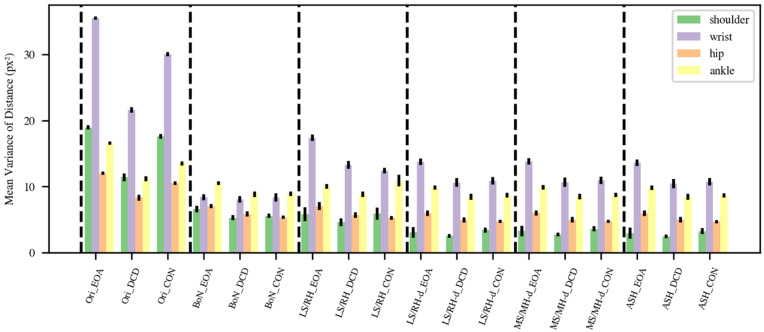
Mean variance of distance between groups. (Ori: original data; BoN: box normalization; LR/RH: left-shoulder right-hip normalization; LS/RH-d: left-shoulder right-hip distance normalization; MS/MH-d: mid-shoulder mid-hip distance normalization; ASH: average shoulder hip normalization).

We subsequently plotted the distance variance for EOA, DCD, and the controls separately in Figure 5 to better comprehend the variability across groups in mean variance of distance. Here, we assume that a good normalization method should (1) preserve the stability of the distance between the shoulders and between the hips within each of the three groups, resulting in minimal shoulder and hip distance variance, and (2) result in larger wrist and ankle distance variance in the EOA group than in the control group [1,2,10,33,34]. The BoN method cannot account for the expected high variability of wrist distance in the EOA group, so we suggest not using this method. The variability of wrist distance in EOA after LS/RH, LS/RH-d, MS/MH-d, or ASH normalization is considerably larger than in the controls and DCD, as may be expected based on EOA phenomenology. Variance in wrist distance in the DCD and the control children shows different results with different normalization methods, which appears to be consistent with our predictions, given that it is difficult to diagnose DCD clinically. Furthermore, ankle variance in the EOA children is larger than in DCD and the control children after LS/RH-d, MS/MH-d, and ASH normalization, as may also be expected based on EOA phenomenology [1,33,34]. Finally, we performed Mann–Whitney U-tests for all three group pairs for ankle and wrist distance variance for each normalization method. The only significant differences found were for mean variance in ankle distance between the EOA and CON groups for the LS/RH-d and ASH normalization methods (LS/RH-d: U = 44, *p* = 0.048; ASH: U = 42, *p* = 0.045). As the goal of these tests was only to identify the best performing method(s), we did not correct for multiple comparisons. To conclude, both the LS/RH-d, and ASH normalization methods performed reasonably well and similarly in our task.

### 3.4. Location Distribution Comparison

**Figure 6:**
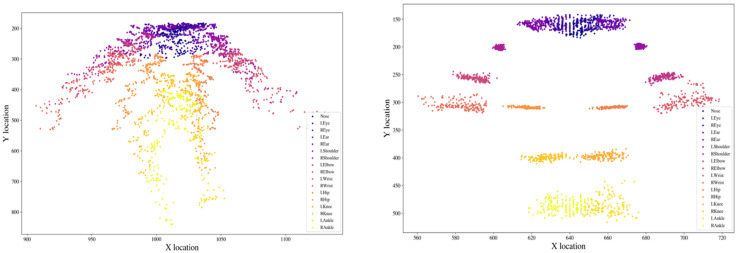
Distribution of the locations of the 17 keypoints before and after LS/RH-d normalization for one segment of a DCD child walking towards/away from the camera. (**Left**): before scaling; (**right**): after scaling.

To better understand what our normalization is accomplishing, we plotted the distribution of the 17 keypoints before and after LS/RH-d normalization for one segment of a DCD child walking towards/away from the camera (See Figure 6). The coordinates on the left (before normalization) are spread widely for each keypoint because when the participant is farther away from the camera, the skeleton’s size becomes smaller in the 2D video frame. After we used the LS/RH-d normalization method, each of the keypoints is localized more concentrated in a small region, resulting in a more stable, robust, and less noisy trajectory. This then allows the derivation of distance-based features and their comparison across different movement disorder groups or use for diagnostic classification.

## 4. Discussion

In this study, we present a framework based on 2D video pose estimation and proposed different normalization methods for skeleton data to enable the quantitative assessment of movement disorders. We found that AlphaPose and PoseTrack based keypoint localization can achieve relatively high confidence in coronal plane 2D recordings, and normalization methods in this framework could be helpful for the calculation of distance- based features.

The high likelihood after cleaning in our dataset from AlphaPose and PoseTrack with pretraining in the MSCOCO dataset supports the potential for quantitative analysis of routine video recordings for assistance in diagnosis and daily monitoring. Our video data, which included 15 children walking freely in a corridor, was captured with a single camera at a resolution of only 1280 × 720 pixels and a frame rate of 25 frames per second. The major reason the confidence cannot achieve values higher than 0.9 after cleaning is that keypoints on the face, such as the nose, eyes, and ears, cannot be seen or have low spatial resolution as people walk away from the camera. Yet, the likelihood findings show that even at low video resolution, the pose estimation model has a high level of reliability, and thus provides a solid platform for further study and demonstrates that it is promising for clinical application.

Assessing movement disorders objectively and accurately is notoriously difficult. Even though machine learning models have been used to predict gait characteristics [35], current models still rely on data from specialized hardware such as optical motion capture devices [36] and inertial measurement units [9]. Deep learning pose estimation approaches have been investigated in clinics for predicting gait parameters [37] and quantifying parkinsonian gait features [38]. However, the approach investigated in this study has not been adequately illustrated or explored, particularly in terms of how to extract distance-based movement features. It is feasible to reconstruct the three-dimensional kinematics of human movement by recording with a 3D capture system with several different cameras [39,40], or with a single RGB-D camera [41]. Previous research has already compared pose estimation from 2D and 3D video data and demonstrated that the 2D skeleton modality with proper preprocessing performed almost as well as the 3D-based method for an action recognition task [42]. In addition, an earlier study compared the validity of 2D and 3D analyses when recording rearfoot walking [43] and found no significant differences between the two approaches on variables commonly examined in rearfoot motion. Furthermore, the comparison of 2D and 3D video analysis during running [44], for angular measurements [45], and for sagittal plane gait assessment [46] supports that implementing 2D video analysis is more reasonable and feasible due to its convenient features compared with the high equipment cost and time required for 3D analysis, and might thus be clinically applicable. Based on this research conclusion, we investigated several normalization methods in 2D from the perspective of convenience and practicality for clinical use. To the best of our knowledge, this is the first study to discuss how normalization may be used to extract distance-based features from 2D skeletons to produce interpretable movement features for clinical research.

This study has some limitations. First, the children with EOA were slightly older than the DCD and the control children. However, as healthy and DCD children grow up, the variability in coordinative movements declines [47,48], and thus the difference in variability in movement between EOA and DCD or the control children would be expected to be even larger if the DCD or the control children had been fully age-matched. Second, our long-term goal is to utilize ubiquitous surveillance cameras (such as mobile phones) to collect data and analyze it in a rapid and easy-to-use way; however, the present approach does not address how the camera’s height and angle impact the results. When the camera moves to different angles, the size of the human skeleton also varies. This could be solved by using a tripod for recording, but this decreases ease of use. Third, the 2D skeleton data still contains some noise after cleaning, and the trajectories of keypoints through time are still not smooth after normalization; filtering could potentially be applied to deal with this. Further, here we express our distance-based measures in pixels and not (yet) in meters. This does allow consideration of variability in such measures, which, as argued, are clinically relevant parameters, but not yet absolute distances. In principle, such absolute distances could be obtained from 2D video frames if a calibration were performed, for example, by measuring the height of the participant and including a standing frame in the video clip. This would allow the expression of pixel sizes in meters. In the current study this was not yet included as we employed existing clinical footage. In further studies when collecting more data prospectively, this calibration step could be added, which would also allow comparison of distance-based features derived from 2D footage with a gold standard measure, such as those derived from 3D optical measurements.

Our normalization framework applied to 2D freehand single camera video recording data demonstrated a promising application for the quantitative assessment of movement disorders in a convenient and objective manner. For future further evaluation of the proposed normalization methods, after inclusion of a calibration step in prospectively collected video recordings, 3D optical systems employing reflective markers could be utilized to establish ground truth. As such, this study is a preliminary step towards quantitative analysis of clinically observed gait using single camera freehand video recording. In addition, if more data is collected, machine learning algorithms could be developed to help differentiate between different movement disorders.

## Figures and Tables

**Table 1:** Likelihood of prediction of keypoint locations.

	Mean	STD
before cleaning	0.817	0.09
after cleaning	0.889	0.02

## Data Availability

The original video datasets are not publicly available for privacy reasons, but the skeleton data after pose estimation presented in this study can be found at: https://drive.google.com/file/d/1hOHnY8qrX4mQ8Lcjc3anOP8qvadCAthR/view?usp=sharing (accessed on 15 May 2022).
